# Multiple Becker’s Nevi in a Female Patient Without Musculoskeletal or Developmental Abnormalities: A Case Report and Review of the Literature

**DOI:** 10.7759/cureus.20712

**Published:** 2021-12-26

**Authors:** Fariba Ghalamkarpour, Nasim Niknezhad, Nazanin Mansourzadeh, Nakisa Niknejad, Farshid Etaee

**Affiliations:** 1 Dermatology, Shahid Beheshti University of Medical Sciences, Tehran, IRN; 2 Pathology, Cancer Institute, Imam Khomeini Hospital Complex, Tehran University of Medical Sciences, Tehran, IRN; 3 Pathology, Hamedan University of Medical Sciences, Hamedan, IRN; 4 Internal Medicine, Yale University, New Haven, USA

**Keywords:** melanocytic nevus, hamartomas, developmental defects, pigmentary disorders, becker's nevus

## Abstract

Becker's nevus (BN) is one of the cutaneous hamartomatous lesions that often presents as an acquired, single unilateral pigmented hairy patch on the upper trunk, scapular region, or upper arm. Hereby we introduce a 13-year-old girl with the unusual manifestation of BN with multiple acquired, pigmented hairy patches for six months without any musculoskeletal or developmental involvements. After carefully reviewing the literature, to the best of our knowledge, this is the first case in this regard in Iran.

## Introduction

Becker's nevus (BN) is one of the cutaneous hamartomatous lesions that often presents as a single unilateral pigmented patch on the upper trunk, scapular region, or upper arm that could be accompanied by hypertrichosis. Occasionally, BN, especially in multiple forms, may accompany some other cutaneous and musculoskeletal presentations such as pectoralis muscle hypoplasia, limb shortening, congenital adrenal hypoplasia, and pectus carinatum. BN is usually unilateral but few reports of bilateral presentation have been reported. BN affects males more than females (M/F: 5) and is acquired in most cases, but inheritance cases with an autosomal dominant pattern have been reported [1]. The main complaint of these patients is a cosmetic issue, and most treatments have focused on cosmetic demands. Patients without non-cutaneous manifestations should be informed about the benign nature of the condition [2]. Microscopic examination of cutaneous lesions reveals epidermal acanthosis, irregular elongation with a fusion of rete ridges, hyperkeratosis, pigmentation of the basal layer, increase in dermal smooth muscle fibers, and melanophages [3]. Here, we report we report a 13-year-old female with multiple BN. 

## Case presentation

**Figure 1:**
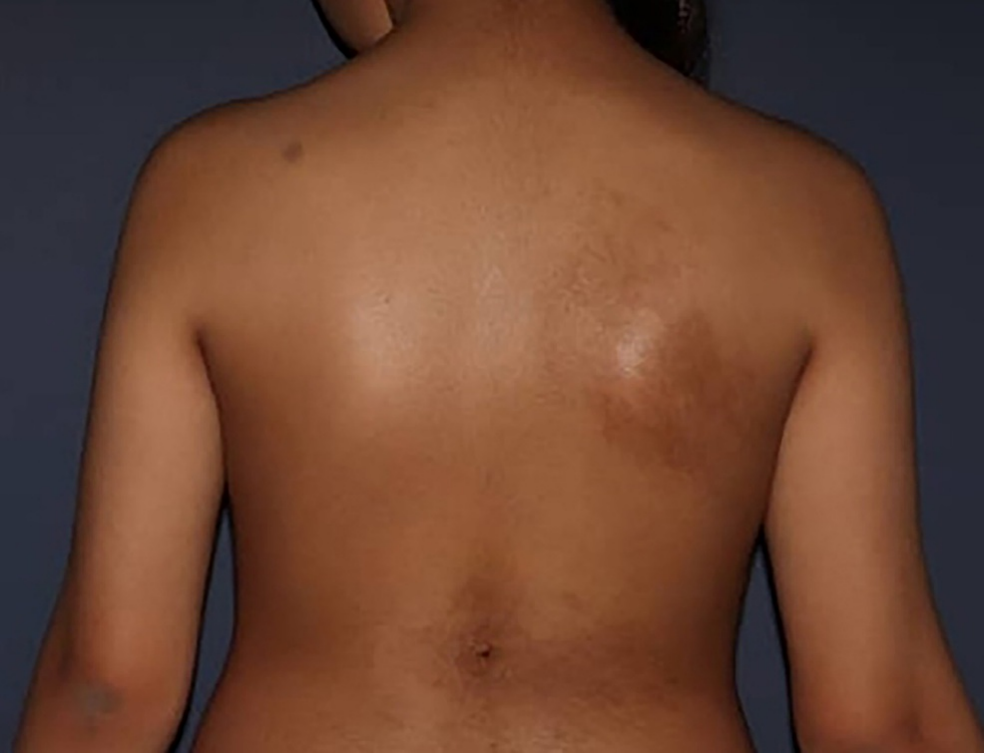
Clinical picture of multiple Becker’s nevi on the back of a 13-year-old girl.

A 13-year-old girl with no significant past medical history was referred to our dermatology clinic with a complaint of two hairy pigmented patches on her back that developed six months ago with no precipitating factor. She was the third child of non-relative parents born by normal vaginal delivery after an uncomplicated perinatal period. Her first clinical examination at birth was normal. Her physical and mental developmental milestones were normal. Physical examination revealed two well-defined, irregular-shaped, dark-brown hairy patches on her back (Figure 1).

**Figure 2:**
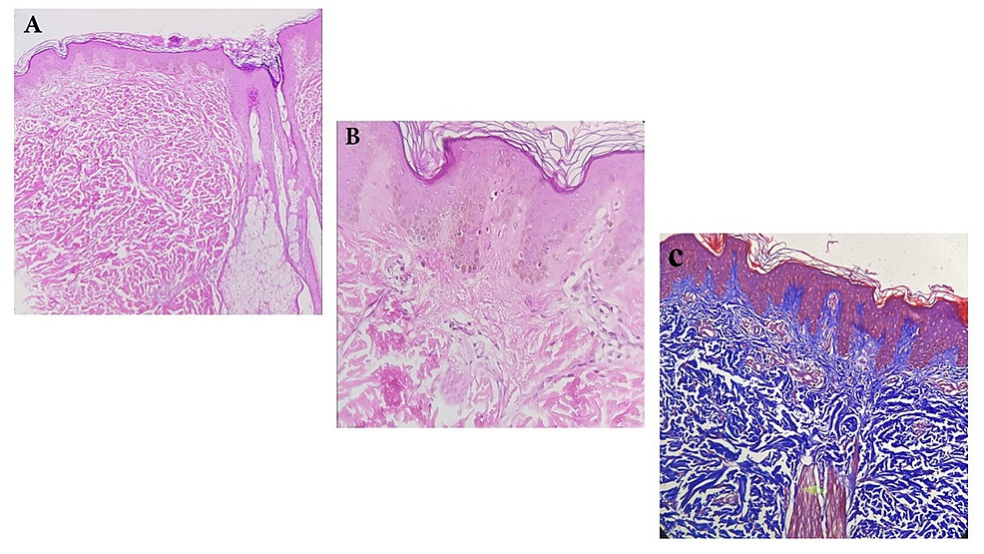
A and B, Histopathology of the Becker’s nevus shows acanthosis of epidermis, elongation, and fusion of rete-ridges. The basal layer of the epidermis is hyperpigmented. Pilar structure with normal appearance is also present. C, Masson trichrome stain that increased collagen fibers with hypertrophic smooth muscle bundle are evident.

Skin biopsy from pigmented hairy patches was performed and microscopic examination showed epidermal papillomatosis, pigmentation of the basal layer, and elongation of rete-ridges (Figure 2).

Mentioned histopathologic findings and clinical features were compatible with BN. Due to the presence of multiple lesions, a complete evaluation of the developmental, musculoskeletal, and genitalia system (to rule out Becker’s nevus syndrome), including total body skin examination, gynecologic examination, and abdominal ultrasound study was done that did not reveal any non-cutaneous abnormalities. Evaluation of the patient’s family history did not show similar cutaneous lesions.

## Discussion

BN is a cutaneous hamartomatous lesion that mostly presents as a single unilateral hyperpigmented patch. Sometimes, BN may be accompanied by hypertrichosis. The most common predilection sites are shoulder, anterior chest, scapula, and back. Most of the cases are acquired and present in childhood or adolescence. BN can present as an isolated lesion or as Becker's nevus syndrome, in combination with mammary hypoplasia, or any other skin or musculoskeletal presentations [4,5]. Treatment is primarily for cosmetic reasons (hyperpigmentation or hair growth) and may include Ruby laser treatment or laser-assisted hair removal. According to the role of androgen receptor density in the pathogenesis of BN, antiandrogens such as spironolactone and topical ﬂutamide have been effective on BN-associated breast hypoplasia and hyperpigmentation [6]. Patients who present with BN should be completely examined for accompanying developmental disorders [7]. The most common developmental disorders that have been documented in these patients are musculoskeletal anomalies such as smooth muscle hamartoma, pectus excavatum, limb shortening, and spina bifida [8]. Other differential diagnoses such as congenital melanocytic nevus, plexiform neurofibroma, and congenital smooth muscle hamartoma should be considered and they should be examined for soft tissue and bony abnormalities [9].

As mentioned, the most common form of BN is a single lesion, and it is significantly more common in males. Based on the literature review, only two female cases of BN have been reported [10,11].

Uncommon presentations such as bilateral BN in female subjects have rarely been reported (Table 1).

**Table 1:** Table 1: Reported cases of multiple Becker's nevi with associated clinical presentation.

	Age	Gender	Presense of developmental disorders	Location of beckers melanosis	Acquired/congenital	reference
1	14	Male	-	Trunk and lower limbs	Acquired/ at age of 12	[1]
2	23	Male	-	Both shoulders and arms	congenital	[2]
3	45	Male	-	Anterior chest	Presentation at adolescence	[7]
4	35	Male	-	Right shoulder and right lower chest	Presentation about puberty	[8]
5	14	Male	-	Upper chest and bilateral upper limb	Acquired /at age of 12	[9]
6	4	Male	-	Bilateral scapular and upper limb	congenital	[12]
7	20	Female	-	trunk	Acquired/At age of 16	[10]
8	15	Female	mental retardation/deafness/short stature/depressed nasal bridge/hypertrophic and hyperpigmented caruncle of both eyes/mitral valve prolapse/skeletal abnormalities (Becker’s Nevus syndrome)	Lower limbs, neck, genitalia	Acquired/ at age of 5	[11]
9	14	Male	-	Back, chest, upper limb	Acquired/ at age of 8	[13]
10	18	Male	-	Back	Acquired/at age of 15	[14]
11	28	Male	-	Trunk, upper limb, lower limb	Acquired/at age of 13	[15]
12	19	Male	Hypoplasia of areola (Becker’s Nevus syndrome)	Trunk, upper limb	-	[16]
13	20	Male	-	Trunk	10	[16]
14	18	Male	-	Back, upper limb, lower limb	-	[17]

It's worth noting that despite observing cases of BN in Iran, this is the first case of multiple BN from this region. Considering the fact that BN is generally uncommon in females, presenting with multiple skin lesions with no skeletal and developmental disorders in a female is extremely rare. In two cases with multiple BN, it was accompanied by skeletal and developmental disorders [11,16]. If systemic work-up rules out the skeletal, developmental, and neoplastic lesions, the treatment protocols are focused on cosmetic procedures, which is very important.

## Conclusions

BN is a cutaneous hamartomatous lesion that mostly presents as a single unilateral hyperpigmented patch that can present with associated hypertrichosis. In our patient, a 13-year-old girl presenting with two hairy pigmented patches for six months without any musculoskeletal or developmental involvements, microscopic examination showed epidermal papillomatosis, pigmentation of the basal layer, and elongation of rete-ridges. It is worth noting that this is the first case of multiple BN from Iran.
